# Root hairs are the most important root trait for rhizosheath formation of barley (*Hordeum vulgare*), maize (*Zea mays*) and *Lotus japonicus* (Gifu)

**DOI:** 10.1093/aob/mcab029

**Published:** 2021-04-20

**Authors:** Emma Burak, John N Quinton, Ian C Dodd

**Affiliations:** Lancaster Environment Centre, Lancaster University, Lancaster, UK

**Keywords:** Rhizosheath, root hairs, root mucilage, maize, barley, *L. japonicus*

## Abstract

**Background and Aims:**

Rhizosheaths are defined as the soil adhering to the root system after it is extracted from the ground. Root hairs and mucilage (root exudates) are key root traits involved in rhizosheath formation, but to better understand the mechanisms involved their relative contributions should be distinguished.

**Methods:**

The ability of three species [barley (*Hordeum vulgare*), maize (*Zea mays*) and *Lotus japonicus* (Gifu)] to form a rhizosheath in a sandy loam soil was compared with that of their root-hairless mutants [bald root barley (*brb*), maize root hairless 3 (*rth3*) and root hairless 1 (*Ljrhl1*)]. Root hair traits (length and density) of wild-type (WT) barley and maize were compared along with exudate adhesiveness of both barley and maize genotypes. Furthermore, root hair traits and exudate adhesiveness from different root types (axile versus lateral) were compared within the cereal species.

**Key Results:**

Per unit root length, rhizosheath size diminished in the order of barley > *L. japonicus* > maize in WT plants. Root hairs significantly increased rhizosheath formation of all species (3.9-, 3.2- and 1.8-fold for barley, *L. japonicus* and maize, respectively) but there was no consistent genotypic effect on exudate adhesiveness in the cereals. While *brb* exudates were more and *rth3* exudates were less adhesive than their respective WTs, maize *rth3* bound more soil than barley *brb*. Although both maize genotypes produced significantly more adhesive exudate than the barley genotypes, root hair development of WT barley was more extensive than that of WT maize. Thus, the greater density of longer root hairs in WT barley bound more soil than WT maize. Root type did not seem to affect rhizosheath formation, unless these types differed in root length.

**Conclusions:**

When root hairs were present, greater root hair development better facilitated rhizosheath formation than root exudate adhesiveness. However, when root hairs were absent root exudate adhesiveness was a more dominant trait.

## Introduction

The rhizosheath is defined as soil that is strongly bound to the root so that it remains adhered after the root has been excavated (McCully, 2005; Ma *et al.*, 2011; Brown *et al.*, 2017; Pang *et al.*, 2017). Rhizosheaths are present in nearly all species of terrestrial plants (Brown *et al.*, 2017) and their ubiquitous nature implies that they are of physiological significance to plants. Indeed, by maintaining close soil bonds at the root surface, the rhizosheath exhibits altered water dynamics compared to the bulk soil, making it more efficient at water retention, protecting the root against drought stress (Young, 1995; Carminati *et al.*, 2010). The rhizosheath has also been attributed with facilitating uptake of nutrients such as phosphorus (Delhaize *et al.*, 2009; Brown *et al.*, 2012; George *et al.*, 2014) and, through microbial associations, nitrogen (Wullstein *et al.*, 1979; Bergmann *et al.*, 2009), as well as ameliorating other abiotic stresses such as aluminium toxicity and extremes of pH (Delhaize *et al*., 2009, 2012; Haling *et al.*, 2010*a*; Lynch and de Leij, 2012). Furthermore, the processes involved in rhizosheath formation create new, or increase the strength of existing, water-stable aggregates (Morel *et al.*, 1991; Albalasmeh and Ghezzehei 2013; Wang *et al.*, 2017), thereby enhancing the stability and structure of the soil.

Rhizosheath formation primarily depends on root traits such as the presence of root hairs (Watt *et al.*, 1994; Haling *et al.*, 2014; George *et al.*, 2014) and root mucilage (Watt *et al.*, 1994; Albalasmeh and Ghezzehei 2013; Carminati *et al.*, 2017). However, soil characteristics such as strength, porosity (Haling *et al.*, 2014) and water content (Watt *et al.*, 1994; Czarnes *et al.*, 2000*a*; Haling *et al.*, 2014) can influence the process. Soil biota associated with the root system can also contribute to rhizosheath formation via microbial-derived mucilage (Watt *et al.*, 1993; Czarnes *et al.*, 2000*b*) and the enmeshing of soil particles by hyphae from mycorrhizal fungi (Degens, 1997; Moreno-Espíndola *et al.*, 2007). However, there has been little attempt to determine whether these physical, chemical and microbiological mechanisms have complementary impacts on rhizosheath formation.

Root hairs are cylindrical subcellular protrusions of the root epidermal cells of most plant species (Dolan and Costa, 2001) and are a key component in rhizosheath formation. Root hair length is often deemed to determine the radial extent of the rhizosheath (Wullstein and Pratt, 1981; Delhaize *et al.*, 2015). Root hairs enmesh soil particles and penetrate soil aggregates, further securing them to the root (Hinsinger *et al.*, 2009; Brown *et al.*, 2012). However, root hair length becomes less correlated with rhizosheath development the longer the root hairs get (Brown *et al.*, 2017) and roots void of root hairs can still form a deficient version of a rhizosheath (Wen and Schnable, 1994; Haling *et al*., 2010*b*, 2014; George *et al.*, 2014). While the mechanisms involved in this process have attracted relatively little attention, it highlights that other root traits are involved in rhizosheath formation.

Root mucilage is also deemed necessary to rhizosheath formation. Root mucilage is a sticky polysaccharide-rich gel-like substance secreted from the root epidermis (Bertin *et al.*, 2003; Akhtar *et al.*, 2018). In rhizosheath formation, hydrated root mucilage permeates soil particles and, when dry, forms hydrophobic bonds between particles (Carminati *et al.*, 2010; Albalasmeh and Ghezzehei, 2013). At low concentrations thin filaments are formed, but with increasing mucilage concentration these filaments can become a network of stable barriers (Carminati *et al.*, 2017). At artificially high quantities, mucilage can form such a comprehensive network of hydrophobic barriers that they impede the passage of water (Benard *et al.*, 2016). All non-woody parts of the root system produce mucilage, though the composition and quantity vary between species (Vančura and Hanzlíková, 1972; Fan *et al.*, 2001). Additionally, root hair mucilage is chemically dissimilar to that produced by the main root (Pena *et al.*, 2012; Muszyński *et al.*, 2015). Determining exudate adhesiveness from wild-type (WT) and root-hairless mutants can discriminate the likely physiological impact of root hair mucilage but has yet to be attempted.

Root trait variation across species affects rhizosheath formation (Brown *et al.*, 2017); however, little is known about the effects of different root types. In cereal crops, two easily distinguishable subcategories of roots can be identified. The larger-diameter seed-derived seminal roots, stem-derived nodal roots and brace roots are termed axile roots, whereas thinner, secondary roots are termed lateral roots. Lateral and axile roots have distinctive functionality. Axile roots have a low mortality rate and are relatively slow-growing, whereas lateral roots tend to constitute most of the root mass, developing quickly in response to available nutrients, but dying off when no longer needed (Drew, 1975; Cahn *et al.*, 1989). Their divergent functions are also evident from their differing morphologies. Lateral roots have a greater capacity for water absorption, whereas axile roots can better transport water and nutrients absorbed by the lateral roots (Varney *et al.*, 1991; Doussan *et al.*, 1998; Carminati, 2013; Ahmed *et al.*, 2015). Even though lateral roots increasingly constitute more of the entire root system with plant development (Drew, 1975; Varney *et al.*, 1991) and the predominant traits of lateral and axile roots vary, their relative impact on rhizosheath formation has not yet been evaluated.

Although root hair and exudate effects on rhizosheath formation are understood individually (Watt *et al.*, 1994), the innate complications of harvesting root exudates from the soil (Oburger and Jones, 2018) and observing root hairs *in situ* (Gyssels *et al.*, 2005; Koebernick *et al.*, 2017) mean that direct comparisons of their relative importance in rhizosheath formation are hard to find. Additionally, root hair properties differ greatly between species (Brown *et al.*, 2017) and can vary between different root types of the same root system (Dittmer, 1949), as does root mucilage composition (Foster, 1982; Peña *et al.*, 2012; Muszyński *et al.*, 2015). We hypothesized that root hairs and root exudates have complementary, additive effects on rhizosheath formation, with variation in these traits determining the effects of different root types. Across three species [barley (*Hordeum vulgare*), maize (*Zea mays*) and *Lotus japonicus* (Gifu)] that differ in root architecture (the model legume *L. japonicus* with a distinct taproot and the cereals barley and maize that form fibrous root systems), the rhizosheaths of root-hairless mutants were compared with their respective WTs with root hairs. To determine intra-species variation in rhizosheath formation, root hair traits and exudates from different root types were investigated.

## MATERIALS AND METHODS

### Genotypes

The three mutants used in this experiment have different origins. The barley root-hairless mutant, aptly named bald root barley (*brb*), is a spontaneous mutation that was discovered during a germination experiment (Gahoonia *et al.*, 2001) with its genetic background in ‘Pallas’, a spring barley cultivar. Conversely, the maize mutant (*rth3*; Wen and Schnable, 1994) and the *L. japonicus* mutant (*Ljrhl1*; Karas *et al.*, 2005) are the result of complex processes to isolate specific genes.

### Growth stage

*Lotus japonicus* seeds were first carefully scoured using sandpaper; maize seeds were initially sterilized using 10 % bleach for 5 min, then rinsed thoroughly with deionized (DI) water. Sterilization was unnecessary for the barley and *L. japonicus* seeds because of low levels of microbial contamination. Once sterilized, the maize seeds, as well as the barley seeds, were germinated in Petri dishes containing two sheets of filter paper (Whatman No. 3) moistened with 5 mL of DI water, then left in the dark for ~3–5 d at room temperature (~20 °C). The *L. japonicus* seeds were germinated in the soil of the filled pots, approximately five seeds per pot, and covered with foil until emergence, at which point the seedlings were thinned out to one shoot per pot. The barley and maize seedlings were transplanted into pots when the radicles were of sufficient length to establish that the root hairs were visually apparent on WT plants and visually lacking in the hairless mutant. Due to the heterozygous nature of the *rth3* seeds, the seedlings were assessed under a dissecting microscope to exclude any that had root hairs present. Since the *brb* and *Ljrhl1* mutants are homozygous mutations, root hair presence did not need to be scrutinized.

After germination and the presence or absence of root hairs was established, the barley and maize seeds were planted 1 cm deep in 4-L pots (22 cm tall, 17 cm top diameter, 13.5 cm bottom diameter), and *L. japonicus* seeds were germinated on 1.5-L pots (10.8 cm tall, 15.5 cm top diameter, 11 cm bottom diameter). The soil used was a sandy loam textured topsoil (Bailey’s of Norfolk; 12 % clay, 28 % silt, 60 % sand and 3 % gravel median diameter 6 mm, no particles >8 mm) packed at an approximate bulk density of 1.3 g cm^−3^. The soil was watered and left to drain until dripping ceased (~48 h). At this time, the weight of each pot was recorded as their drained capacity. Each pot was rewetted to drained capacity every second day, allowing the wetting–drying cycles necessary for rhizosheath formation (Carminati *et al.*, 2010; Albalasmeh and Ghezzehei, 2013). *Lotus japonicus* plants transpired less water so were rewetted every 2–3 d. Water was withheld for up to 3 d before harvest to facilitate the effective excavation of the rhizosheath. The plants were cultivated in a walk-in controlled environment room equipped with metal halide lamps (photosynthetic photon flux density at pot height 253 µmol m^−2^ s^−1^; HQI-BT 400 W/D Pro, Osram, Germany) and set at a 12-h photoperiod. Temperature was set at 24 °C during the day and 19 °C at night.

Each experiment comprised 20 replicates of each genotype, with five harvested on each of four occasions. For barley and maize these were 5, 10, 15 and 20 d after the seedlings were transplanted into pots. For the much slower-growing *L. japonicus*, plants were harvested 32, 44, 58 and 71 d after seed germination.

### Quantifying rhizosheath weight

**Fig. 1.**
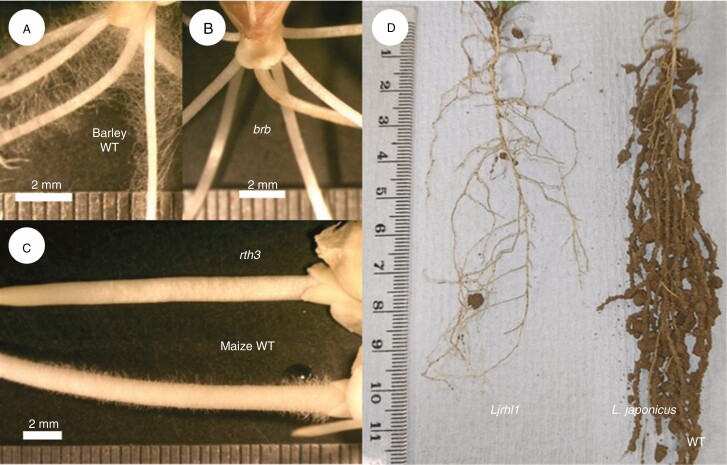
The barley (A, B), maize (C) and *L. japonicus* (D) mutant genotypes without root hairs and their WTs with root hairs. The *L. japonicus* genotypes (D) have been directly removed from the soil and possess their intact rhizosheaths.

**Fig. 2.**
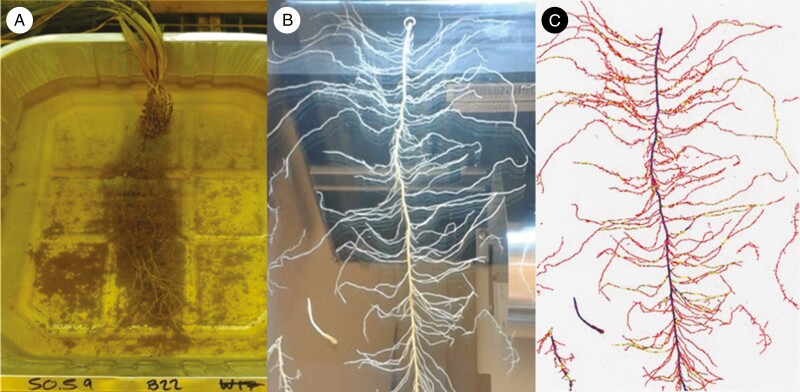
A freshly excavated WT barley root system soaking in a metal dish of water (A). Once the soil had been gently removed the roots were spread out on a flatbed scanner (B). The resulting images were processed in WinRHIZO (C); here the axile (blue) and lateral (red and yellow) roots are easily distinguishable by a 253.6-µm diameter threshold.

At harvest, the whole plant was systematically extracted from the soil, whilst minimizing soil disturbance to retain root–soil contact (Fig. 1D), as previously described (Young, 1995; Veneklaas *et al.*, 2003; Ma *et al.*, 2011; Haling *et al.*, 2014; Pang *et al.*, 2017). The entire root system was then soaked in a metal dish filled with water (Fig. 2A) and gently agitated until the rhizosheath separated from the root. Larger aggregates were fragmented using a paint brush and a wash bottle. Immediately after extraction, all root material was sealed, moist, in a plastic bag and stored at 4 °C for later analysis. The dish was then placed in a drying oven at 105 °C to drive off excess water and the rhizosheath weight was recorded after a constant weight was established.

### Root measurement

Root systems were scanned within 5 d of being extracted from the soil, to avoid any visual physical degradation. Roots were placed in a clear plastic tray with a thin film of water and splayed to avoid as much overlap as possible (Fig. 2B). It was sometimes necessary to separate a single root system into multiple scans. Images were produced as .jpgf files in 8-bit greyscale and at a resolution of 600 d.p.i. for barley and *L. japonicus*, and 400 d.p.i. for maize. For barley and maize, images were captured using an Epson Perfection V700 scanner; for *L. japonicus* an Epson Expression 11000XL Pro scanner with a transparency unit was used. Root length was analysed using WinRHIZO (2013e, Regent Instruments). Debris with a width:length ratio <4 was excluded.

In this paper, the terms ‘axile roots’ and ‘lateral roots’ are defined as shoot/seed- and root-derived roots, respectively (McCully, 1999; Hund *et al.*, 2009). For *L. japonicus*, axile roots include the taproot and any root derived from the taproot. Axile and lateral roots were distinguished by their diameter using the method developed by Hund *et al.* (2009). In WinRHIZO, root length was grouped in units of diameter. The unit increments were determined by the size of one pixel: 31.7 µm for 600 d.p.i. and 63.5 µm for 400 d.p.i. The root data for all harvests were then combined and a second-degree polynomial model was fitted to the data using the loess smoothing function in MATLAB (R2017b) to reveal the two peaks of lateral roots and axile roots (details can be found in Supplementary Data Fig. S1). The diameter that best distinguishes the two root types is represented as the lowest point in the trough between two peaks: 253.6 µm for barley, 635.0 µm for maize and 380.4 µm for *L. japonicus* (Fig. 2C). The maize threshold is consistent with previous findings of Hund *et al.* (2009), which puts the threshold for their maize cultivar at 650 µm. Absolute growth rates of both axile and lateral roots were calculated by dividing the average growth per harvest by the number of days after germination and were expressed as an average across all four harvests.

### Root hair measurements

Barley and maize WT genotypes were germinated and grown using the same methods and environments as described above. The slow growth rate of *L. japonicus* was not compatible with experimental constraints. Wild-type seeds were grown in 1.5-L pots (dimensions as before) containing sandy loam-textured topsoil as described above. After 3 weeks of growing under well-watered conditions, the roots were removed from the soil and gently washed to remove soil particles whilst keeping the root hairs intact. The roots were then photographed at ×25 magnification using a camera (GX Optical GXCAM-H5) attached to a dissecting microscope. For barley, four axile roots and six or seven lateral roots were selected from each plant. The lateral roots were photographed every centimetre from the tip and the axile roots were photographed every 4 cm. For maize, four or five axile roots (representative of axile, primary and crown roots) and four to six lateral roots were selected from each plant. Both axile and lateral roots were photographed every 4 cm from the tip. Each species had four replicates. The subsequent images were then converted to 8-bit greyscale using Gimp 2.6.0. The brightness and contrast were also altered to counter the differing brightnesses of the images.

To establish average root hair length, ten root hairs were measured in each image using the line-measuring function in ImageJ (Brown *et al.*, 2017). To establish root hair density, the total length of both the root hairs and the origin root were measured using WinRHIZO. Due to an illumination gradient, the root–background threshold had to be manually adjusted for each image. Root hair length density (RHLD) was calculated by dividing the total length of root hairs by the length of the origin root segment.

### Soil adhesion assay

While many studies have sought to determine root exudate composition (Bertin *et al.*, 2003; Walker *et al.*, 2003; Naveed *et al.*, 2017), this method was adapted from Akhtar *et al.* (2018) to establish functional differences between exudates of species, genotypes and root types collected under standardized conditions. Seeds were germinated directly into Rockwool kept in a reservoir of 100 % Hoagland solution. Four seeds of each of the barley and maize genotypes were used, and again *L. japonicus* was excluded from this experiment due to its slow growth rate. When the roots were deemed long enough to reach the hydroponic solution (23 d) the plants were transferred to 5-L aerated buckets filled with Hoagland solution (50 % strength for barley and 100 % for maize).

After a further 25 d of growth, root exudates were harvested in 50-mL Falcon tubes filled with DI water and suspended from the buckets’ plastic covers, two per bucket. Some axile roots were isolated in one of the tubes and some lateral roots in the other, leaving the rest of the root system with access to the nutrient solution. A control tube containing only DI water was also placed in half of the buckets to ensure no cross-contamination occurred. The roots were left in the tubes for 4 d, topping up the DI water as needed.

After removing the tubes, the contents were filtered through filter paper (Whatman No. 3) to remove large root particles and then frozen. After lyophilizing the samples to remove the water, the exudates from each replicate were consolidated into one. Exudates from each root were diluted into four aliquots (50, 25, 10 and 1 µg/5 µL) and applied in triplicate onto a dry nitrocellulose membrane sheet (Amersham Protran 0.45 µm, Fisher Scientific, UK). Each 5-µL drop was placed within a 1-cm grid. The nitrocellulose sheets were then placed in aluminium dishes and left to air-dry for at least 1 h before being rewetted with DI water and covered with ~1 cm of air-dried soil sieved to ≤500 μm. The nitrocellulose sheets were left to dry overnight, with their soil covering. When dry, excess soil was shaken off and the nitrocellulose sheet submerged in DI water for 2 s, twice, to remove any extra soil not adhered. Each sheet was then replaced in the metal tray and loosely covered to prevent contamination whilst it was left to air-dry.

Images of the soil-adhered sheets were made the Epson Expression scanner mentioned above at 1200 d.p.i. and in 8-bit greyscale. The soil adhered to each spot was analysed using ImageJ. The mean greyscale value was used to determine how much soil adhered to the nitrocellulose sheet. The mean greyscale value was then converted into soil weight using a calibration curve developed, using the same technique, with drops of gum tragacanth (G1128, Sigma–Aldrich) at a dilution of 50 µg/5 µL. Varying amounts of soil were adhered to small pieces of nitrocellulose sheet, weighing the sheets before and after applying soil.

### Statistical analysis

Analysis of covariance (ANCOVA) assessed the differing abilities of the genotypes to bind soil, with rhizosheath as the main effect and root length as the covariate. ANCOVA was also used to determine if there were differences between the root length and root hair properties of the different root types, with root hair length as the main effect and root length as the covariate. Two-way analysis of variance tested whether the relative root lengths of the genotypes statistically differed. Absolute root growth rate (AGR) was calculated as follows:


$$\begin{array}{l} AGR=\frac{L_{2}-L_{1}}{t_{2}-t_{1}} \end{array}$$

where L and t are total root length and growth duration at sequential harvests 1 and 2. Data from multiple harvests were used to calculate AGR, so root length was plotted against time and the slope of the trend line was regarded as AGR.

To assess the impact of each root type on rhizosheath formation, a linear model was fitted to the data of each species using the MATLAB function fitlm. To avoid the issue of autocorrelation, each predictor variable was modelled individually, so three models per species were created, with genotype, axile and lateral root lengths as predictor variables and rhizosheath weight as the response variable. The models calculated effect sizes, which were compared to establish the relative contributions of each root trait to rhizosheath formation. The impacts of species, genotype, root type and exudate saturation on soil adherence were assessed using four-way analysis of variance.

## RESULTS

**Fig. 3.**
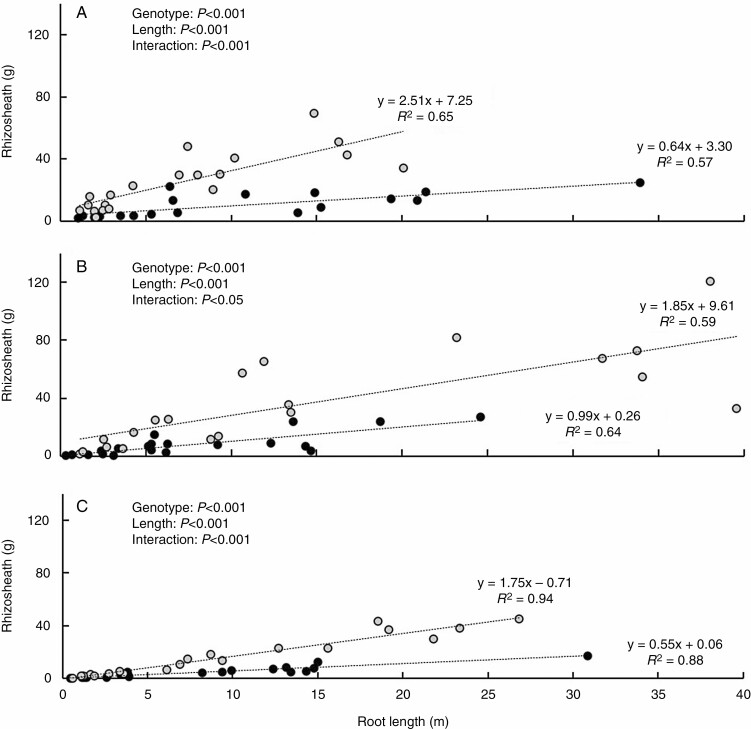
Rhizosheath weight plotted against total root length for barley (A), maize (B) and *L. japonicus* (C). Black symbols represent individual plants of the root-hairless mutants *brb* (A), *rth3* (B) and *Ljrhl1* (C), while grey symbols represent their respective WT. Each panel shows all plants from all four harvests. A linear model was fitted to each genotype, represented by the dashed lines and corresponding equation. All trend lines have a *P* value <0.001 and *R*^2^ > 0.57. *P* values are from ANCOVA.

As expected, rhizosheath weight significantly (*P* < 0.001) increased with root length, but in all three species the WT genotypes bound significantly (*P* < 0.001) more soil than their respective root-hairless mutants (Fig. 3). When comparing the slopes of the rhizosheath versus root length regression lines, barley showed the biggest genotypic difference, with WT binding 3.9-fold more soil than *brb* (Fig. 3A). The *L. japonicus* WT bound 3.2-fold more soil than *Ljrhl1* (Fig. 3C) and the maize WT bound 1.8-fold more soil than *rth3* (Fig. 3B). Despite their lack of root hairs, all three root-hairless mutants formed a rhizosheath, albeit to a lesser extent than their WT counterparts. These genetic differences in rhizosheath formation increased with increasing root length, as indicated by a significant (*P* < 0.05) genotype × root length interaction in each species.

**Fig. 4.**
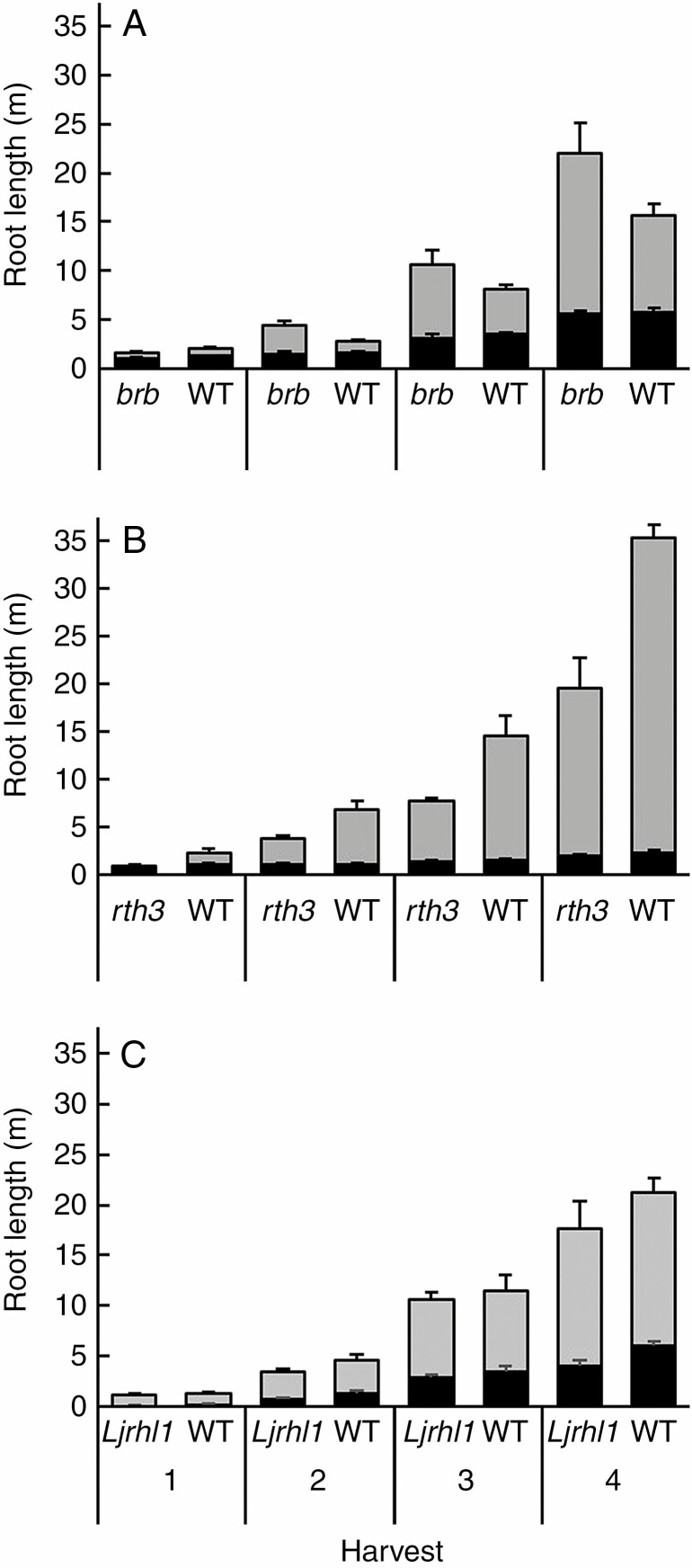
Total root length apportioned into axile (black) and lateral (grey) contributions per harvest for barley (A), maize (B) and *L. japonicus* (C). Error bars are equal to 1 standard error for each root type.

Total root length varied between species and genotypes (Fig. 4, Table 1). The maize WT consistently produced a more abundant root system than *rth3* (*P* < 0.001). The *L. japonicus* WT tended to do the same, though the increase was not significant (*P* = 0.23). However, for barley, *brb* seemingly compensated for the lack of root hairs by proliferating their lateral roots to achieve a significantly greater root length than the barley WT (*P* < 0.05). Thus, when considering all the root-hairless mutants, there was no consistent effect of lacking root hairs on root length.

**Table 1.** Two-way ANOVA results for total root length, axile root length and lateral root length for each species. *P*-values in bold represent significant results

		Total root length		Axile root length		Lateral root length	
		*F* value	*P* value	*F* value	*P* value	*F* value	*P* value
Barley	Genotype	6.062	**0.019**	0.853	0.363	8.883	**0.005**
	Harvest	55.865	**0.000**	91.118	**0.000**	37.705	**0.000**
	Interaction	1.957	0.140	0.172	0.915	2.446	0.082
Maize	Genotype	37.195	**0.000**	4.189	**0.049**	39.360	**0.000**
	Harvest	105.234	**0.000**	15.467	**0.000**	109.979	**0.000**
	Interaction	8.283	**0.000**	0.683	0.569	9.422	**0.000**
*L. japonicus*	Genotype	1.479	0.233	9.908	**0.004**	0.390	0.536
	Harvest	50.747	**0.000**	70.551	**0.000**	43.171	**0.000**
	Interaction	0.425	0.737	2.626	0.067	0.130	0.942

Absolute root growth rates differed statistically between the root types for all genotypes (*P* < 0.05) within species, except for the barley WT (*P* = 0.137). Axile roots grew universally slower than the lateral roots, but their AGR did not vary much between the root-hairless mutants and their WT. The total length of the axile roots of barley and maize increased by 28.45 ± 0.02 and 8.47 ± 0.11 cm d^−1^, while *L. japonicus* increased at a slower rate of 0.13 ± 0.02 cm d^−1^. Thus, the barley genotypes did not produce significantly different lengths of axile roots (*P* = 0.36; Table 1), though the maize and *L. japonicus* WT produced significantly greater axile root length than their respective root-hairless mutants (*P* < 0.05 and *P* < 0.01 for maize and *L. japonicus*, respectively), likely the result of root hairs aiding seedling establishment. Growth rates of lateral roots far exceeded those of the axile roots and showed more genotypic variation. For barley, lateral root growth rates of *brb* and its WT were 97.0 and 57.1 cm d^−1^, respectively, resulting in *brb* producing significantly more lateral root length than its WT (*P* < 0.01). In maize, growth rates were 111.8 and 205.74 cm d^−1^ for *rth3* and its WT, respectively, resulting in *rth3* producing significantly more lateral root length than its WT (*P* < 0.001). Again, *L. japonicus* was the slowest-growing and had the least genotypic variation; lateral root growth rates for *Ljrhl1* and its WT were 0.33 and 0.36 cm d^−1^, respectively, with no significant genotypic effect on root length (*P* = 0.54). Lateral roots constituted 65.8, 88.7 and 73.8 % of the root system in barley, maize and *L. japonicus*, respectively. Their more prolific growth rate means that lateral roots constitute the bulk of the root system, and strongly influence the genotypic variation in root length.

**Fig. 5.**
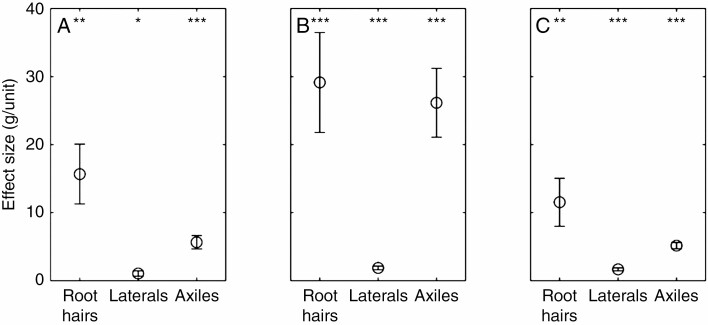
Estimated effect on rhizosheath formation of a 1-unit increase in root type as calculated by a linear regression model for barley (A), maize (B) and *L. japonicus* (C). The unit for root hairs is a binary increase from absence to presence and the units for axile and lateral roots are for a 1-m increase in root length. Error bars are equal to 1 standard error. *P < 0.05, **P < 0.01, ***P < 0.001.

As expected, all root types had a significant positive effect on rhizosheath formation (Fig. 5). The presence of root hairs had the single biggest impact on rhizosheath formation across all species. For maize the presence of root hairs resulted in a mean of 29.2 ± 7.3 g increase in rhizosheath weight across the whole root system. For barley and *L. japonicus* the magnitude of the root hair effect was less, with a mean increase in rhizosheath weight of 15.7 ± 4.4 and 11.5 ± 3.5 g, respectively. Increasing axile root length growth (by 1 m) had the next biggest influence on rhizosheath weight across all species. Again, barley and *L. japonicus* responded similarly, with rhizosheath weight increasing by 5.6 ± 0.1 and 5.1 ± 0.5 g m^−1^ of axile root growth, respectively. In maize, axile root growth increased rhizosheath weight by 26.2 ± 5.1 g. Although lateral roots were the fastest-growing root type, they had the smallest impact on rhizosheath weight, resulting in a 1.0 ± 0.4, 1.9 ± 0.3 and 1.6 ± 0.2 g m−1 rhizosheath increase for lateral root growth of barley, maize and *L. japonicus*, respectively. Thus, rhizosheath weight increased with both axile and lateral root length, but the presence of root hairs had the greatest impact on rhizosheath formation.

**Fig. 6.**
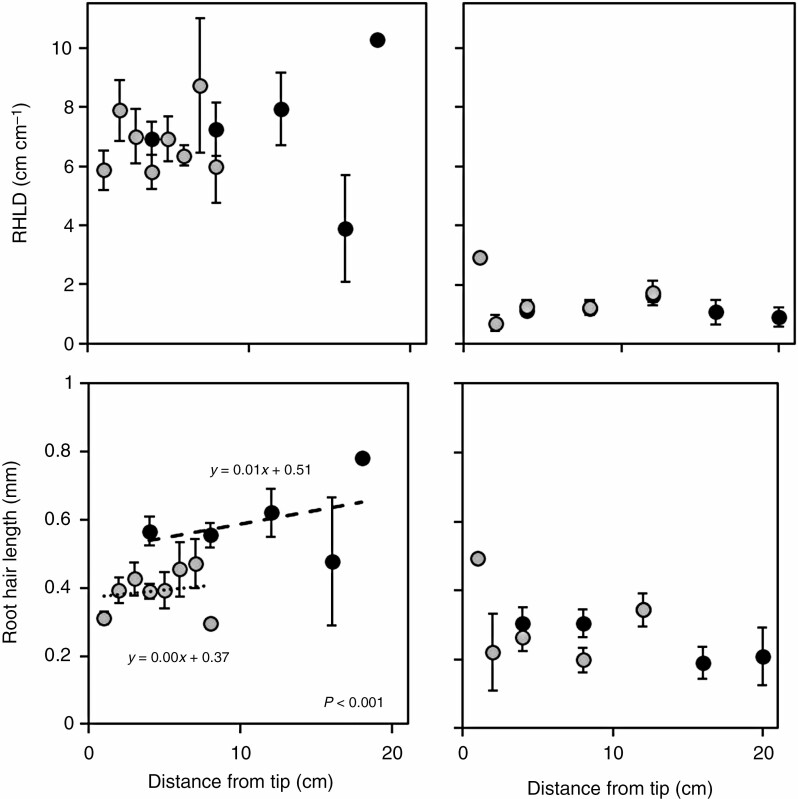
Root hair length density (A, C) and length (B, D) versus distance from the root tip in barley (A, B) and maize (C, D). Grey symbols represent lateral roots and black symbols represent axile roots. Error bars are equal to 1 standard error. Linear regressions were fitted when the root types differed significantly.

Root hair length and RHLD were significantly correlated for both barley (*R*^2^ = 0.54, *P* < 0.001) and maize (*R*^2^ = 0.36, *P* < 0.001). Root hair length density did not differ between the axile and lateral roots of either barley or maize, nor did RHLD change with increasing distance from the root tip (Fig. 6). Similarly, root hair length on lateral and axile roots was comparable in maize and did not differ with distance from the tip. However, barley axile roots produced 26 % longer root hairs in comparison with their lateral roots (calculated by the intercept of the regression lines in Fig. 6). In comparing species, barley produced significantly longer root hairs (2-fold, *P* < 0.001) and at a greater RHLD (6-fold, *P* < 0.001) than maize. Since both root hair measurements did not change with increasing distance from the root tip for either species, it can be assumed that all ages of roots display a similar number and length of root hairs.

**Fig. 7.**
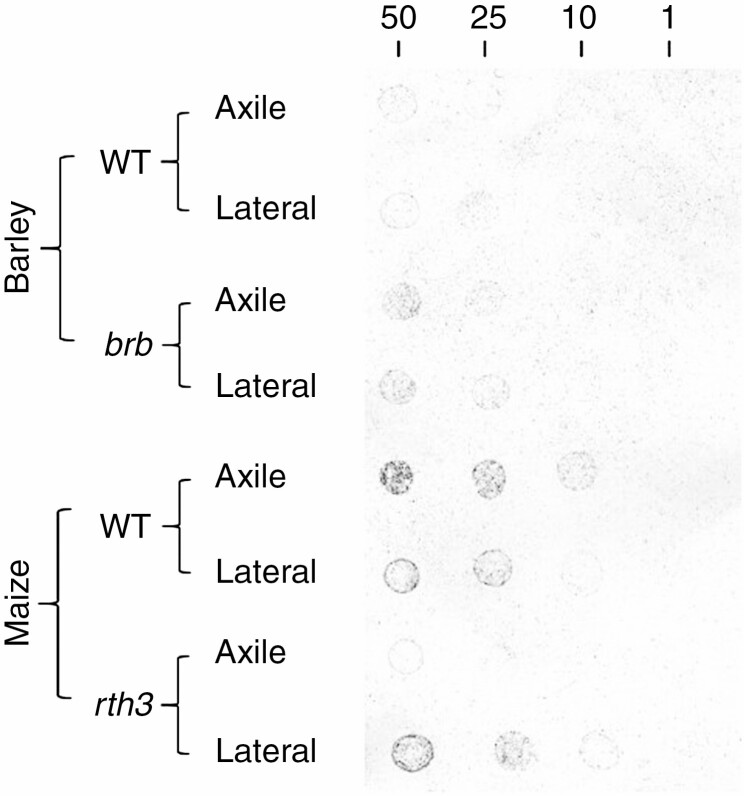
Scanned images from the soil adhesion assay. The drops are distributed in a 10-mm square grid. The dilution of exudates in DI water descends from left (50 µg/5 µL) to right (1 µg/5 µL), with a single droplet comprising 5 µL.

**Fig. 8.**
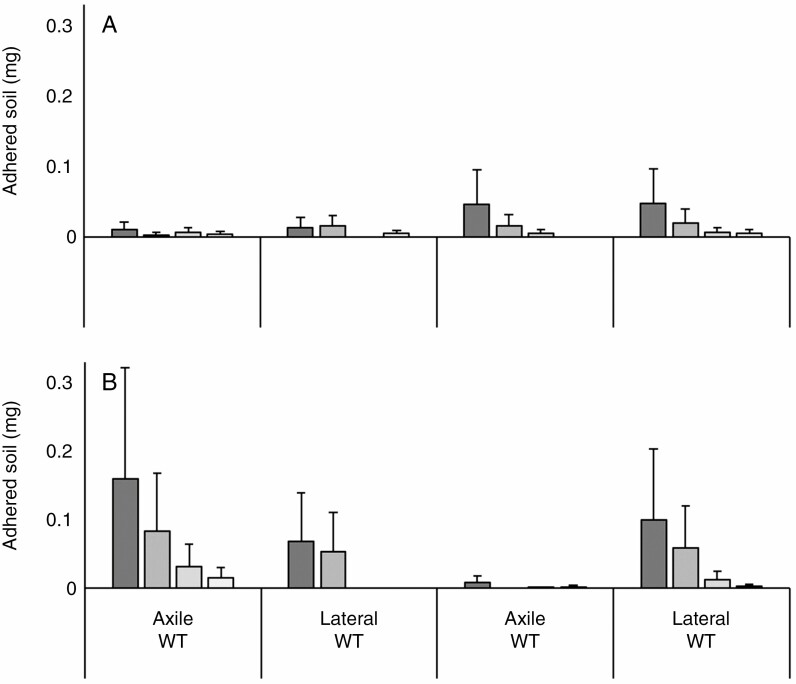
Amount of soil adhering to a nitrocellulose membrane spotted with root exudates collected from the axile and lateral roots of the barley (A) and maize (B) genotypes. The bars are coded in shades of grey to reflect the mucilage saturation of the droplet, from darkest, 50 µg/5 µL, through 25 and 10 µg/5 µL, to lightest, 1 µg/5 µL. Error bars are equal to 1 standard error.

Soil adhered to the root exudates placed on nitrocellulose sheets (Fig. 7) with significant variation in the adhesive capacities of exudate from different species (*P* < 0.05; Table 2) and genotypes (*P* < 0.05). For barley, the exudates from *brb* showed a greater capacity to bind soil than its WT (Fig. 8A). Exudates from the barley WT axile and lateral roots were both relatively ineffective at binding soil, since soil adhesion was only just above background levels across the whole dilution scale. For both *brb* and WT, exudate adhesiveness of the different root classes did not differ (Fig. 8, Table 2). Maize root exudates were significantly (*P* < 0.05) more effective at binding soil than barley root exudates. Root exudates from WT maize axile roots adhered the most soil of all the roots tested, in contrast to the *rth3* axile root exudates, which were barely above background levels (Fig. 8B). Root exudates from the maize lateral roots showed that *rth3* and its WT had a similar capacity to bind soil. Overall, maize root exudates were more adhesive than barley roots, and the effect of root hairs varied with species.

**Table 2.** Analysis of variance table for the soil adhesion assay. *P*-values in bold represent significant results

	Sum of squares	d.f.	Mean square	*F* value	*P* value
Species	0.027	1	0.027	5.156	**0.026**
Genotype	0.049	2	0.025	4.780	**0.011**
Root type	0.006	1	0.006	1.144	0.288
M. saturation^a^	0.012	3	0.004	0.771	0.513
Error	0.456	88	0.005		
Total	0.536	95			

^a^Mucilage saturation in droplet (50, 25, 10 and 1 µg/5 µL).

## Discussion

While many root traits (including root number, root type and root age) could influence soil adhesion, both root hairs (McCully, 2005; Moreno-Espíndola *et al.*, 2007; Haling *et al*., 2010*a*, *b*; Brown *et al*., 2012, 2017; Delhaize *et al*., 2012, 2015; George *et al.*, 2014; Adu *et al.*, 2017; Pang *et al.*, 2017) and root exudates (Watt *et al.*, 1994; Albalasmeh and Ghezzehei 2013; Carminati *et al.*, 2017) are widely recognized to greatly affect rhizosheath formation. However, the relative contribution of these traits to rhizosheath formation has yet to be considered. Measuring rhizosheath formation of several root-hairless mutants and their respective WTs (Fig. 3) as well as comparing different root types (Fig. 4), and relating them to root hair (Fig. 6) and mucilage properties (Fig. 8), demonstrated that when root hairs were present their properties were the most dominant factor determining rhizosheath mass (Fig. 5). Since the combined effect of exudates and root hairs varied between species (root hairs enhanced rhizosheath formation by 3.9-, 1.8- and 3.2-fold for barley, maize and *L. japonicus,* respectively), our hypothesis of additive complementary effects of the two mechanisms was disproved. However, in the root-hairless mutants exudate adhesiveness became dominant, with variation in the chemical composition of root mucilage, to some extent, compensating for the absence of root hairs physically enmeshing soil particles.

Root hairs significantly enhance rhizosheath formation (with root-hairless mutants binding less soil than their WT in three different species; Fig. 3) by enmeshing soil particles and physically anchoring them to the root as well as increasing the area of the root for soil to be chemically bound to (Hinsinger *et al.*, 2009; Brown *et al.*, 2012). Without root hairs, soil can only be chemically bound to the main roots. Although increased total root length of older plants (Figs 3 and 4) can eventually compensate for the lack of root hairs in rhizosheath formation, genotypic differences between WTs and root-hairless mutants also increased with root length. Thus, the additional root length needed by the mutants to compensate for their lack of root hairs also increases. Growing new roots with root hairs has a disproportionately greater impact on rhizosheath formation than the same length of new root without root hairs. However, as in previous studies (Wen and Schnable, 1994; Haling *et al*., 2010*b*, 2014; George *et al.*, 2014) root-hairless mutants still bound some soil, indicating that both physical (root-hair enmeshment of soil particles) and chemical (root exudates adhering to soil particles) mechanisms contribute to rhizosheath formation.

Longer root hairs generally increase rhizosheath size (Wullstein and Pratt, 1981; Haling *et al*., 2010*a*, 2013; Brown *et al.*, 2012; Delhaize *et al*., 2012, 2015; George *et al.*, 2014; Adu *et al.*, 2017) and can explain species differences in rhizosheath development. In the present study barley root hairs (0.6 and 0.4 mm for axile and lateral roots, respectively) were longer than maize root hairs (0.3 mm for both axile and lateral roots, Fig. 6), which agrees with previous measurements (Gahoonia *et al.*, 2001; Zhu *et al.*, 2005) and explains why differences in rhizosheath formation between the WT and hairless mutant were so much greater in barley than maize (Fig. 3). Additionally, increased root hair development of WT barley meant it was 1.5 times more effective at binding soil than a WT maize root system of equivalent accumulated length. As root hairs extend radially from the root, the distances between root hairs will increase, and thus root hair extension has limited impact on increasing rhizosheath formation because the interaction between the intersecting soil and root hairs weakens with distance from the root. Since further increases in root hair length have increasingly limited effects on rhizosheath formation beyond a threshold of 0.28 mm (Brown *et al.*, 2017), other traits, such as root hair distortion and density and variations in root exudates, likely become more important (Brown *et al.*, 2017; Pang *et al.*, 2017).

Although the impact of RHLD on rhizosheath formation is not known, root hair length and density can disproportionately affect other root functions, such as nutrient uptake (Itoh and Barber, 1983; Zygalakis *et al.*, 2011). Additionally, roots can compensate for the reduced rhizosheath-forming capacity of short root hairs by increasing their density (Adu *et al.*, 2017). For both barley and maize, RHLD showed similar differences between axile and lateral roots (Fig. 6). Similarly, barley root hairs were denser (4-fold more root hairs per millimetre) than maize root hairs. Considering that barley root hairs were also longer than maize root hairs makes it difficult to distinguish whether it was the increased length or the increased density that enhanced their ability to bind soil; however, an argument can be made that the greater density supported the increased length, allowing a stable rhizosheath to continue to form as the root hairs extended from the root. Although root hair length and density can respond similarly to environmental (Watt *et al.*, 1994; Haling *et al.*, 2010*a*) and endogenous factors, such as auxin, which promotes both root hair initiation and elongation (Ma *et al.*, 2001), root hair length and RHLD are not always correlated (Haling *et al.*, 2010*a*; Nestler *et al.*, 2016; Adu *et al.*, 2017). Therefore, to fully test this hypothesis, genotypes with root hairs of the same length but with different densities would need to be compared.

Compared with the thicker, slower-growing axile roots involved in structural support and nutrient transport (Varney *et al.*, 1991; Doussan *et al.*, 1998; Carminati, 2013; Ahmed *et al.*, 2015), the thinner and longer lateral roots (Drew, 1975; Cahn *et al.*, 1989) associated with nutrient and water uptake are assumed to be more effective at forming a rhizosheath, since their putative functions overlap. However, in this study, when root hairs are the same length and at the same RHLD (as in maize; Fig. 6C, D), axile and lateral roots contributed similarly to rhizosheath formation. Although the linear regression model suggested that axile root growth affected rhizosheath formation more than lateral root growth (Fig. 5), axile roots grow much slower than lateral roots (Fig. 4; Drew and Saker, 1975; Cahn *et al.*, 1989; Pagès and Pellerin, 1994). This dynamic gives the illusion that axile roots have a greater impact on rhizosheath formation, when in fact their slower growth rates (95 % less than lateral roots) are commensurate with their apparent effect (Fig. 5) on rhizosheath formation (axile root growth seemingly had 93 % greater effect than lateral roots). Therefore, in the time taken for axile roots to increase by one unit length, total root length and its consequent rhizosheath would have increased proportionally more as a result of the much greater lateral root growth rate (Fig. 4). Although root hair properties of axile and lateral roots of *L. japonicus* were not measured, the similar quantitative effects of axile root growth rate (63 % slower than their lateral roots) and rhizosheath formation (68 % greater than lateral roots) suggests that *L. japonicus* root hair properties do not differ between root types (as in maize). Thus, their relative contribution to rhizosheath formation depends on their growth rate compared with the overall increase in root system size and not an increased affinity for rhizosheath formation. Likewise, when root types differ in their root hair development (as in barley; Fig. 6B), root hair properties influence the root’s ability to form a rhizosheath. Unlike maize, barley axile roots affected rhizosheath formation disproportionately to their growth rates (Fig. 5). Although barley lateral roots grew 63 % faster than axile roots, the latter had 82 % more effect on rhizosheath formation because they had longer (by 26 %) root hairs (Fig. 6). Thus, the longer root hairs on the barley WT axile roots were more efficient at binding soil than the shorter root hairs on the barley lateral roots. So, the capacity to form a rhizosheath depends less on root type and more on their respective root hair properties.

**Fig. 9.**
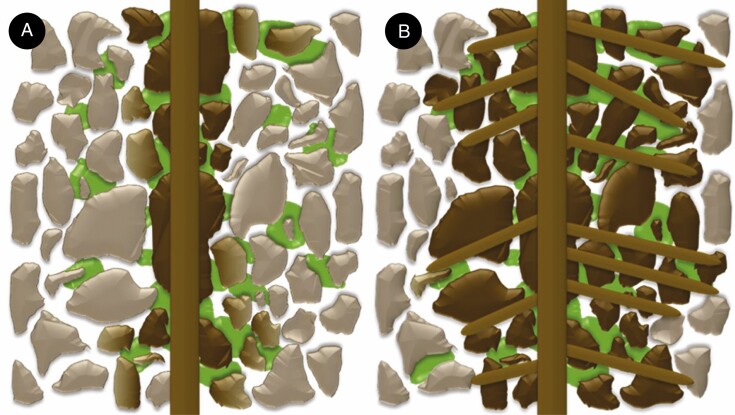
A conceptual representation of the contributions that root hair and mucilage traits make to rhizosheath formation. For both images the soil particles that are not faded represent the rhizosheath soil, bound to the root. For roots without any root hairs (A) only soil particles that are in direct contact with the main root are bound as a rhizosheath. If the root produces a more adhesive mucilage, then it binds more soil particles (represented by the particles with a graduated fade). The presence of root hairs (B) increases the root surface area for the soil particles to bind to, resulting in a more prominent rhizosheath. Longer and denser root hairs (right side of B) increase the radial extent of the rhizosheath more than when root hairs are shorter and less dense (left side of B).

That root-hairless mutants bind some soil, albeit much less, shows that root traits other than root hairs (such as root exudates) also determine rhizosheath development (George *et al.*, 2014; Haling *et al.*, 2014). However, their significance in root-hairless mutants has hitherto not been evaluated. Overall, maize exudates were far more adhesive than barley exudates. Similarly, Naveed *et al.* (2017) found that barley root exudates initially act to weaken the soil, whereas maize roots more actively bind the soil, with these differences attributed to altered chemical composition of the exudate. When root hairs were present, the increased adhesiveness of the maize WT did not outweigh the benefits of increased root hair development, as it bound 1.4 times less soil than the barley WT. Root exudate adhesiveness cannot completely compensate for shorter root hairs between species, but becomes more important when root hairs are absent (Figs 3, 6 and 8). The increased adhesiveness of *rth3* root exudates meant it was 1.5 times more effective at binding soil than *brb* root systems of the same length. While adhesive root exudates can aid rhizosheath formation, and even determine its extent in the absence of root hairs, the presence and abundance of root hairs is the strongest driver of rhizosheath development (Fig. 9).

Root exudate adhesiveness is assumed to relate to the concentration of polysaccharides in the exudate, one of the main components understood to influence rhizosheath formation (Morel *et al.*, 1991; Piccolo and Mbagwu, 1999; Czarnes *et al.*, 2000*b*; Carminati *et al.*, 2017; Galloway *et al.*, 2018), though not all substances in root exudates are adhesive (Read *et al.*, 2003; Akhtar *et al.*, 2018). Root exudates comprise a complex combination of substances, including those that have no apparent function except as waste products of internal metabolic processes (Walker *et al.*, 2003) and those that facilitate nutrient uptake (e.g. phytosiderophores) and root lubrication (Bertin *et al.*, 2003). Organic acids have been linked to the dispersion of soil particles, thereby decreasing soil aggregation (Oades, 1984; Goldberg *et al.*, 1990; Read *et al.*, 2003; Naveed *et al.*, 2017) and increasing the availability of root-accessible phosphate and micronutrients (Hinsinger, 2001). While both barley and maize exudates comprise predominantly organic acids, barley exudates contain about twice as much as maize, whereas maize exudates contain about twice as much polysaccharide as barley (Naveed *et al.*, 2017). The greater propensity of maize root exudates to bind soil has previously been linked to increasing soil water retention, suggesting that the composition of root exudates can influence plant water and nutrient uptake strategies (Pang *et al.*, 2017; Ndour *et al.*, 2020).

Since an increasing number of studies indicate that rhizosheath is important in enhancing plant nutrient acquisition (Watt *et al.*, 1994; Ma *et al.*, 2011; Brown *et al.*, 2012), the relative carbon costs to the plants in its creation should be considered. Despite continuous production and high turnover of barley root hairs (McElgunn and Harrison, 1969), comparing root respiration of WT and root-hairless mutants of *Arabidopsis* revealed they had limited metabolic costs (Bates and Lynch, 2000), although future comparisons should be made in the same species. In contrast, the metabolic costs of root exudation are highly variable but can comprise up to half of all below-ground carbon supply (reviewed in Lynch and Ho, 2005). While root hairs may be seen as a more carbon-efficient mechanism of enhancing rhizosheath formation than exudation, diffusion of the latter into the bulk soil can cause secondary adhesion (beyond the physical dimensions of the root hairs) and stimulate microbial activity (Zhang *et al.*, 2020) to substantially extend rhizosheath diameter. These effects may be particularly important in promoting rhizosheath longevity in species such as barley that undergo cortical senescence of older axile roots (Schneider *et al.*, 2017), leading to the epidermis being shed. Since our experiments used young actively growing plants (<20 d old for barley and maize and <71 d old for *L. japonicus*), the relative contributions of root processes that minimize respiratory costs (e.g. root cortical aerenchyma formation in maize, cortical senescence in barley) to rhizosheath longevity were not explicitly assessed, but these may account for variable relationships between rhizosheath size and root length reported in the literature (Pang *et al.*, 2017). While the metabolic costs of different processes affecting rhizosheath formation merit further study and are borne by one crop generation, their longevity and persistence in the soil after root senescence and death (Williams and Weil, 2004) create pores in the soil that can benefit the following crop by allowing root exploration of deeper soil layers in search of moisture (White and Kirkegaard, 2010; Hodgkinson *et al.*, 2017).

### Conclusions

The presence of root hairs significantly enhances rhizosheath formation, but root mucilage has an important role when root hairs are absent. Differences in physical root hair properties (length and density) most readily explain variation in rhizosheath formation between different species, mutants and root types, with further work required to disentangle the relative contributions of root hair length and density.

## SUPPLEMENTARY DATA

Supplementary data are available online at https://academic.oup.com/aob and consist of the following. Figure S1: root diameter thresholds (vertical lines) distinguishing lateral from axile roots of barley, maize and *L. japonicus*.

mcab029_suppl_Supplementary_Material
