# Supplementary material for: Root hairs are the most important root trait for rhizosheath formation of barley (Hordeum vulgare), maize (Zea mays) and Lotus japonicus (Gifu)
Source: Ann Bot. 2021 Apr 20;128(1):45–57. doi: 10.1093/aob/mcab029 (PMC8318254; doi:10.1093/aob/mcab029)
Supplement: mcab029_suppl_Supplementary_Material [file mcab029_suppl_supplementary_material.docx]

# Supplementary information

## Root diameter thresholds.


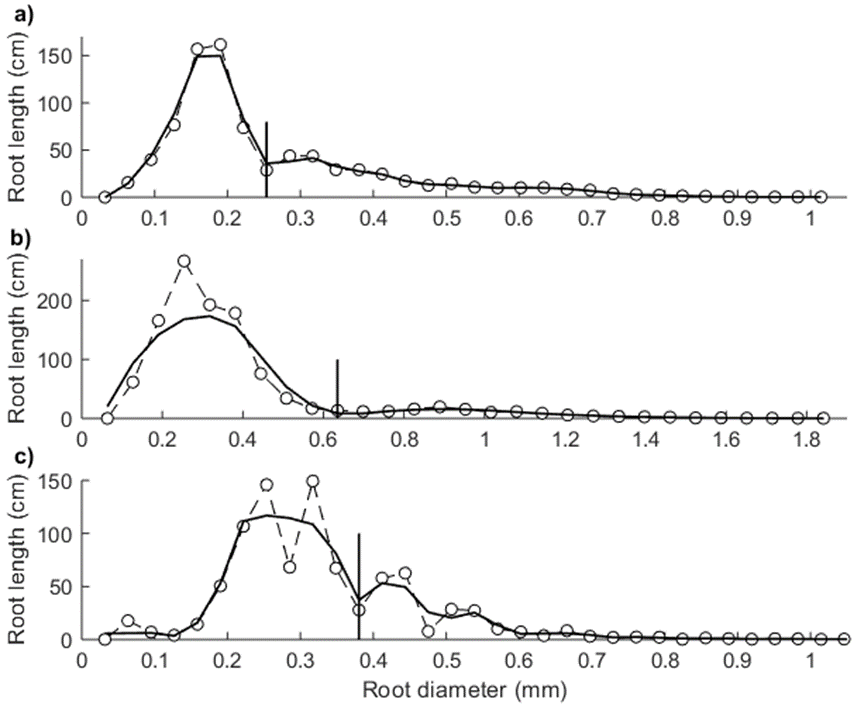


Figure S1. Root diameter thresholds (the vertical lines) distinguishing lateral from axile roots of barley (a), maize (b), and *L. japonicus* (c). Each symbol represents the total root length in the diameter class. The dashed line depicts the actual data and the solid line represents a second degree polynomial model. Each species had a different diameter distribution, so weightings were adjusted accordingly with a neighbourhood weighting of 20% for barley, 40% for maize, and 10% for *L. japonicus*. The diameter thresholds were also assessed visually in WinRHIZO to ensure they accurately distinguished the root types.
